# The Role of Fecal Calprotectin in Patients with Systemic Sclerosis and Small Intestinal Bacterial Overgrowth (SIBO)

**DOI:** 10.3390/diagnostics10080587

**Published:** 2020-08-13

**Authors:** Beata Polkowska-Pruszyńska, Agnieszka Gerkowicz, Karol Rawicz-Pruszyński, Dorota Krasowska

**Affiliations:** 1 Chair and Department of Dermatology, Venerology and Pediatric Dermatology, Medical University of Lublin, Staszica 16, 20-400 Lublin, Poland; agerkowicz@wp.pl (A.G.); dor.krasowska@gmail.com (D.K.); 2 Department of Surgical Oncology, Medical University of Lublin, Radziwiłłowska 13, 20-080 Lublin, Poland; krpruszynski@gmail.com

**Keywords:** calprotectin, small intestinal bacterial overgrowth, SIBO, systemic sclerosis

## Abstract

Fecal calprotectin (FC) is a quick, cost-effective, and noninvasive test, which is used to diagnose patients with active inflammatory bowel diseases (IBD). Recent studies suggest the possible predictive role of FC in the diagnosis of small intestinal bacterial overgrowth (SIBO) in patients with systemic sclerosis (SSc). This study aimed to assess the predictive value of FC in SSc patients and its’ possible use as a SIBO marker. A total of 40 SSc patients and 39 healthy volunteers were enrolled in the study. All subjects completed questionnaires evaluating gastrointestinal symptoms, FC measurements, and lactulose hydrogen breath test (LHBT) assessing SIBO presence. After rifaximin treatment, patients with SIBO underwent the same diagnostic procedures. Significantly higher FC values were observed in the study group compared to controls (97 vs. 20 μg/g; *p* < 0.0001) and in SSc patients diagnosed with SIBO compared to SSc patients without SIBO (206 vs. 24 μg/g; *p* = 0.0010). FC turned out to be a sensitive (94.12%) and specific (73.68%) marker in the detection of SIBO in patients with SSc (AUC = 0.82, 95% CI = 0.66–0.93; *p* < 0.0001). Our study suggests the potential value of FC in SSc in detecting gastrointestinal impairment and its promising role as an additional diagnostic tool for SIBO.

## 1. Introduction

Calprotectin is a heterodimer of 36.5 kDa belonging to the S100 family and characterized by calcium and zinc binding, a central hinge, N- and C-terminal domains, and solubility in 100% ammonium sulphate at neutral pH [1,2,3]. Its antimicrobial role is based on binding transition metals, thereby preventing their utilization by invading microbes. During infection and inflammation, increased calprotectin release is observed, mainly within neutrophils but also within monocytes and macrophages in lower quantities [4,5,6]. The antimicrobial role of calprotectin has been demonstrated against *Escherichia coli*, *Klebsiella* sp., *Staphylococcus aureus*, *Listeria monocytogenes*, and *Salmonella enterica* as [7]. Additionally, as a damage-associated molecular pattern molecule (DAMP), it has the ability to activate toll-like receptors, which is part of the innate immune response [8].

Apart from other body fluids, calprotectin can be found in stool samples. Fecal calprotectin (FC) levels are dependent on the inflammatory state of the intestinal mucosa and the accumulation of neutrophils responsible for its production. This correlation has paved the way for FC in gastrointestinal symptom diagnostics. Elevated FC levels has been demonstrated in patients with inflammatory bowel disease (IBD) [9]. FC distinguishes IBD from functional bowel disorders at 63–100% sensitivity and 80–100% specificity rates [10,11,12,13,14,15,16,17]. Furthermore, recent studies have indicated FC utility in IBD, irritable bowel syndrome (IBS), and nonorganic bowel disease differentiation [18]. Additionally, FC can serve as therapeutic follow-up marker in disease monitoring, and its increased levels may allow early endoscopic intervention [18]. Furthermore, FC measurement is an easy, cost-effective, quick, noninvasive, and reproductible diagnostic procedure [7]. However, false-positive results of FC may be observed during treatment with anti-inflammatory agents (NSAIDS) and proton pump inhibitors (PPI), and therefore their administration should be ceased before conducting FC measurement [18]. The FC values in healthy individuals should not exceed 50 μg/g of feces. However, the normal FC values depend on the patient’s age and are much higher in pediatric population, particularly in children aged under five years [7,19].

Systemic sclerosis (SSc) is a chronic, connective tissue disease of autoimmune origin and characterized by multiorgan malfunction [20]. Approximately, 90% of SSc patients are at risk of developing gastrointestinal complications [21]. The progressive fibrosis within gastrointestinal tract causes hypomotility as well as the stasis of intestinal content, which promote extensive bacterial colonization. This quite frequently results in small intestinal bacterial overgrowth (SIBO) syndrome development, which is described as overgrowth of bacteria to over 10^5^ CFU/mL (colony forming units per mL of jejunal juice) or the presence of atypical flora [22,23,24,25,26]. One of the first-choice diagnostic procedures for SIBO is lactulose hydrogen breath test (LHBT) [27]. Due to progressive skin fibrosis and the development of microstomia, SSc patients find it difficult to exhale through a mouthpiece and sometimes even through a facemask. In these cases, LHBT becomes a more challenging test. Therefore, the search for other possible diagnostic markers of SIBO remains crucial. Some recent studies have suggested possible value of fecal calprotectin in detecting SIBO in SSc patient population [28,29,30].

The aims of the present prospective study were as follows: (i) determine the FC levels in patients with SSc and healthy controls; (ii) establish the correlation between SSc clinical characteristics and levels of FC; and (iii) evaluate the correlation between FC levels and digestive symptoms as well as gastrointestinal involvement, including the presence of SIBO.

## 2. Patients and Methods

A total of 40 patients with SSc diagnosis based on the 2013 classification criteria for systemic sclerosis by the American College of Rheumatology/European League against Rheumatism collaborative initiative and 39 healthy controls were enrolled in the study in the Department of Dermatology, Venerology, and Pediatric Dermatology, Medical University of Lublin, Poland [31]. All participants joined the study in the period from December 2018 to February 2020. Written informed consent was obtained from all study subjects before enrolment to the study. The study was approved by the Local Ethics Committee: KE-0254/238/2018 (25/10/2018)), and data was analyzed from a prospectively maintained database.

All study subjects enrolled in the study underwent the same diagnostic procedures, including gastrointestinal symptom questionnaires, lactulose hydrogen breath test (LHBT), and fecal calprotectin measurement. Initially, all study participants completed a gastrointestinal symptom questionnaire, which classified the complaints—diarrhea, constipation, abdominal pain/discomfort, flatulence, abdominal tenderness, nausea, vomiting, dysuria, tenesmus, fever, general illness, reflux, dysphagia, and early satiety—in the range from 0 (no symptoms) to 3 (very often). Moreover, the subjects completed the UCLA Scleroderma Clinical Trial Consortium GIT 2.0 (UCLA SCTC GIT 2.0) questionnaire, which consists of seven items—reflux, distention/bloating, diarrhea, fecal soilage, constipation, emotional well-being, and social functioning—scored from 0.00 to 3.00, except diarrhea and constipation, which are scored from 0.00 to 2.00 and from 0.00 to 2.50, respectively. The total score of gastrointestinal symptom severity is the summation of all scales (except constipation) [32].

All study participants underwent LHBT after preparation (restricted diet, avoidance of certain drugs and endoscopic procedures, fasting period of at least 12 h). Exhaled air sample was analyzed using Gastrolyzer (Bedfont, Maidstone, Kent, UK). Basal air sample was assessed at fasting and then after oral administration of lactulose (20 g in 200 mL of sterile water) at intervals of 20 min during the period of 3 h. An increase of 20 parts per million (ppm) of expired hydrogen between the maximum reading and the basal value equaled a positive result.

Fecal calprotectin was measured using CALEX^®^ Cap Stool Extraction Device and Cobas^®^ 6000 analyzer series (Roche, Switzerland) according to the manufacturer’s instructions in the study and control group. As PPI and NSAID use appears to be associated with significantly elevated fecal calprotectin, the patients enrolled in the study were instructed to discontinue PPIs and NSAIDs at least one week prior to FC measurement. The cut-off level for abnormal FC values was estimated above 50 μg/g. We evaluated the biochemical, demographic, and clinical characteristics of SSc patients, including measurements of erythrocyte sedimentation rate (ESR; mm/h), C-reactive protein (CRP; mg/L), total protein (g/dL), albumin (% of total protein), C3 complement (g/L), C4 complement (g/L), creatine kinase (CK, IU/L), rheumatoid factor (IU/mL), NT-proBNP (pg/mL), leukocytes (K/uL), alanine transaminase (ALT, U/L), aspartate transaminase (AST, IU/L), neutrophils (K/µL), antibody status (anticentromere (ACA), antitopoisomerase I (anti-Scl70), and anti-RNA polymerase III antibodies), median age, weight, body mass index (BMI), median SSc duration, SSc subsets, and the presence of gastrointestinal comorbidities.

All study subjects with SIBO diagnosis underwent rifaximin therapy (400 mg 3 times daily for 10 days). One month after the therapy, the patients underwent LHBT to determine the eradication of SIBO, filled the gastrointestinal symptom questionnaires, and had FC values measured. Out of the 19 SSc patients with SIBO diagnosis, 15 patients attended the check-up; four patients were lost from the follow-up due to illness or withdrawal of willingness to participate in the study.

We compared the values of FC between SSc patients and healthy controls, FC levels between SIBO-positive and SIBO-negative SSc patients, and FC before and after eradication treatment with rifaximin in SSc patients. We tried to establish the connection between FC levels and certain demographic, clinical, and biochemical characteristics in both SIBO-positive and SIBO-negative SSc patients.

The statistical analyses were conducted using MedCalc 15.8 (MedCalc Software, Ostend, Belgium). For group comparison involving binary data (or for several subgroups), we used either the chi square test or Fisher’s exact test. Comparisons involving continuous data were performed using (1) Student’s *t*-test when distribution of variables was normal and (2) Mann–Whitney test in other cases. The results were regarded as significant when the *p* value was less than 0.05. Furthermore, receiver operating characteristic (ROC) curve was constructed to examine the predictive value of the FC level in detecting specific clinical conditions (occurrence of SSc, presence of SIBO using glucose H_2_/CH_4_ breath test, etc.); the overall diagnostic accuracy of this test was assessed using the area under the ROC curve.

## 3. Results

The study cohort included 39 women (97.5%) and one man (2.5%) with a median age of 62 years (range: 34–78), weight of 65 kg (range: 50–99), and BMI of 24.9 (range: 19.8–34). The median disease duration was 10.5 years (range: 1–30). The control group included 36 women (92.3%) and three men (7.7%) with a median age of 61 years (range: 33–77), weight of 69 kg (range: 53–110), and BMI of 26.4 (range: 19–38.4). Both groups were comparable in terms of sex, age, and weight. All patient characteristics, including the levels of fecal calprotectin, are presented in Table 1.

In the study group, 19 patients (47.5%) were diagnosed with SIBO, whereas SIBO was diagnosed in five cases in the control group (12.8%; *p* = 0.0008). Based on LGBT, eradication of SIBO was achieved in 73.3% of SSc patients. We did not perform gastrointestinal symptom questionnaires, LHBT, and fecal calprotectin measurement after eradication treatment in the controls with SIBO diagnosis due to the unrepresentative number of the group.

Significantly higher FC values were observed in the study group compared to controls (97 vs. 20 μg/g; *p* < 0.0001). FC levels were significantly higher in SSc patients diagnosed with SIBO compared to SSc patients without SIBO (206 vs. 24 μg/g; *p* = 0.0010). The calprotectin level was lower in SSc patients with eradicated SIBO (156 vs. 137 μg/g). However, the obtained result was statistically insignificant. Detailed characteristics and comparison of the FC level between the control and study group (including measurements prior and after treatment) are presented in Table 1.

Among SSc patients without SIBO diagnosis, a positive correlation between FC level and age (rho = 0.505; *p* = 0.0275), CRP value (rho = 0.564; *p* = 0.0120), neutrophils (rho = 0.781; *p* = 0.0001), and leukocyte level (rho = 0.580; *p* = 0.0093) was observed, whereas a negative correlation was observed between this marker and alanine transaminase (rho = −0.567; *p* = 0.0114). A positive correlation between FC levels and neutrophils was also observed among SSc patients with SIBO diagnosis (rho = 0.561; *p* = 0.0191). Detailed data on Spearman’s rank correlation between fecal calprotectin levels and selected demographic, clinical, and laboratory variables depending on the presence of SIBO are given in Table 2. In both SIBO-positive and SIBO-negative SSc patients, no statistically significant correlations were found between the FC level and selected gastrointestinal symptoms assessed in both the questionnaires. Out of the five SSc patients with gastroesophageal reflux disease (GERD), two of them were negative for SIBO with median FC values remaining within normal values (46 ug/g), whereas the remaining three patients with GERD who were positive for SIBO had highly elevated FC levels (371.67 ug/g).

In SSc patients, positive correlation was found between calprotectin and CRP levels (rho = 0.330; *p* = 0.0483). In addition, the FC level assessed after eradication correlated with the level of the quality of life index depending on skin symptoms (rho = −0.711; *p* = 0.0317). Detailed data on Spearman’s rank correlation between FC levels and selected demographic, clinical, and biochemical variables are presented in Table 3.

**Figure 1:**
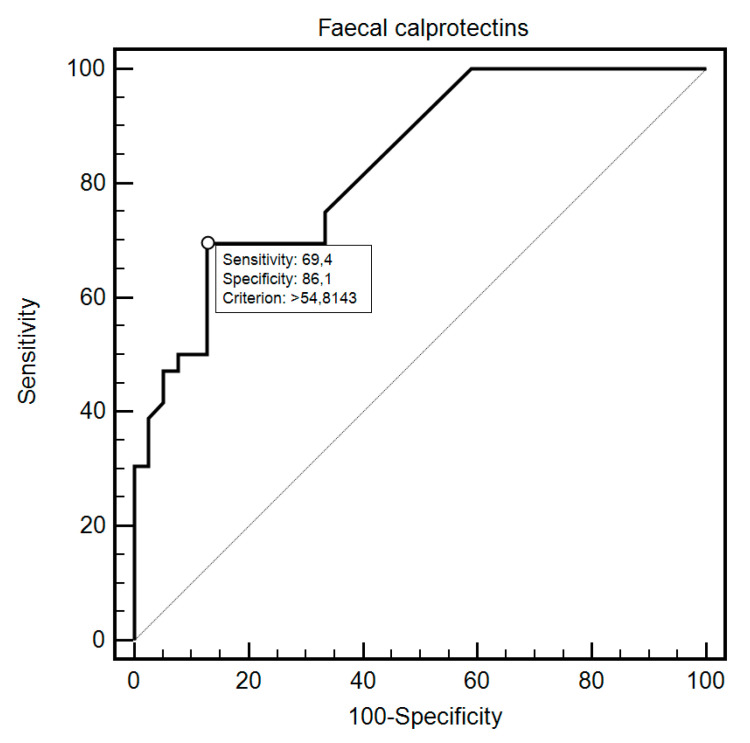
Receiver operating characteristic (ROC) curve illustrating the diagnostic utility of fecal calprotectin in SSc detection.

**Figure 2:**
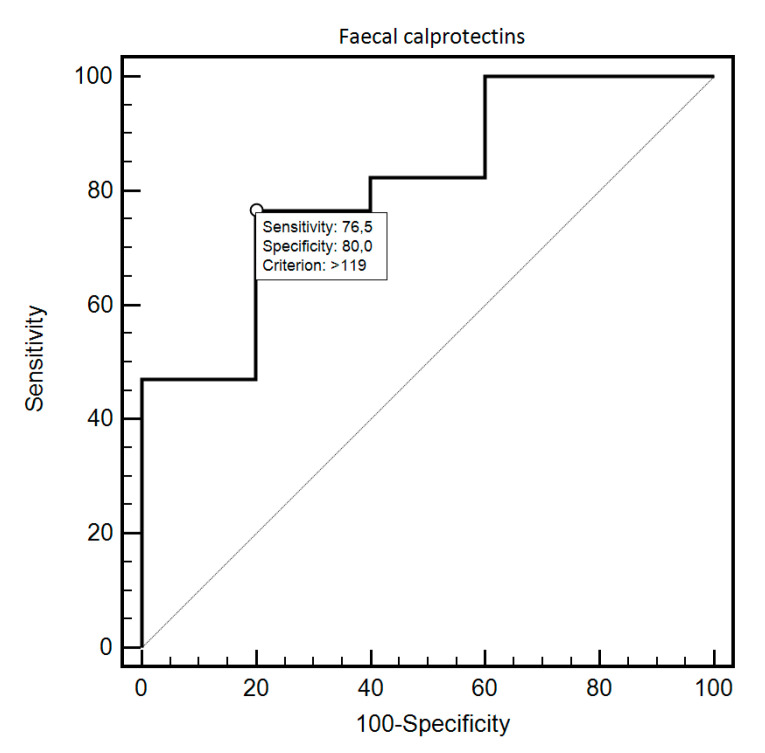
ROC curve illustrating the diagnostic usefulness of fecal calprotectin in SSc detection in individuals with SIBO.

**Figure 3:**
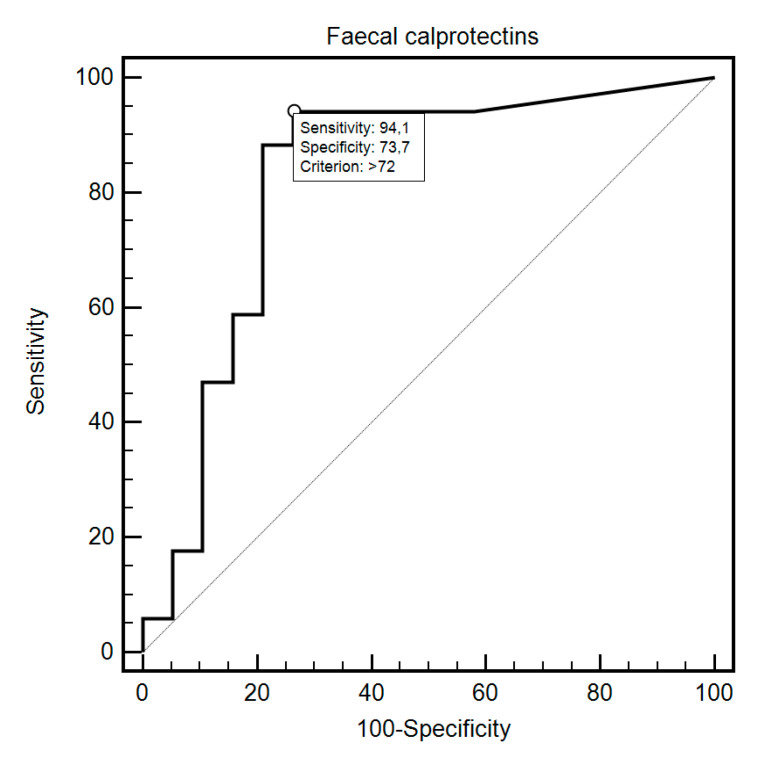
ROC curve illustrating the diagnostic usefulness of fecal calprotectin in SIBO detection in SSc patients.

FC was able to differentiate patients from the control and study group with 69.44% sensitivity and 87.18% specificity (AUC = 0.83, 95% CI = 0.73–0.91; *p* < 0.0001; Figure 1). Additionally, FC showed 76.47% sensitivity and 80% specificity in detecting SSc in patients with SIBO (AUC = 0.81, 95% CI = 0.59–0.94; *p* = 0.0054; Figure 2). FC was also 94.12% sensitive and 73.68% specific in the detection of SIBO in patients with SSc (AUC = 0.82, 95% CI = 0.66–0.93; *p* < 0.0001; Figure 3). FC also proved to be a sensitive (75.86%) and specific (66.67%) marker to detect a specific disease subtype (dcSCC) (AUC = 0.72, 95% CI = 0.54–0.86; *p* = 0.0477). FC showed 90% sensitivity and 61.54% specificity in ACA antibody detection (AUC = 0.74, 95% CI = 0.56–0.88; *p* = 0.0095). Detailed FC characteristics, including its usefulness in detecting SIBO, predicting eradication efficacy, and differentiating different clinical conditions, are presented in Table 4.

## 4. Discussion

Approximately 90% of SSc patients develop variously intensified fibrosis within the gastrointestinal tract (GIT) with subsequent motility disturbances [21]. GIT distortions may have a severe impact on quality of life, morbidity, and eventually mortality. Taking this into account, early identification of the GI involvement is a vital step in SSc management and quality of life improvement [33,34]. Quick diagnosis gives healthcare professionals the means to decelerate disease progression and improve the patient’s prognosis [35]. After esophagus, the small bowel is the second most commonly affected organ in SSc [36]. Up to 50% of patients with esophageal disease and 20% of those with small bowel involvement remain asymptomatic [37,38]. Intestinal hypomotility originating from vasculopathy, smooth muscles atrophy, and fibrosis promotes extensive bacterial colonization and may lead to SIBO [21,24,39].

SIBO remains a diagnostic challenge due to lack of standardization, limited validation, cost-effectiveness, complexity, and low accessibility of small intestinal aspirate culture. Considering acceptable specificity, LHBT is an attractive alternative, although it is characterized by lower sensitivity compared to duodenal aspiration and culture [27,40,41]. In our study, we used LHBT to determine SIBO presence. Most researchers, however, use glucose hydrogen breath test (GHBT) for SIBO diagnosis in SSc patients [39]. Nevertheless, LHBT has been suggested as a first-line diagnostic option by the Gastrolyzer manufacturer. Additionally, in spite of the moderately better diagnostic efficacy of GHBT, we chose LHBT to avoid underdiagnosis and undertreatment of patients with SIBO.

Nevertheless, the search for an ideal diagnostic method or an additional SIBO marker to enhance LHBT value requires further research. Some studies have reported the potential diagnostic role of short-chain fatty acids in small intestine aspiration, unconjugated bile acids in serum, urinary excretion of *p*-aminobenzoic acid or indican, and a 72 h test for fecal fat evaluation. However, their clinical usefulness has not been clearly confirmed [42].

In our study, significantly higher FC values were observed in the study group compared to controls (97 vs. 20 μg/g; *p* < 0.0001). This indicates frequent gastrointestinal involvement in SSc patients. FC levels were significantly higher in SSc patients diagnosed with SIBO compared to SSc patients without SIBO (206 vs. 24 μg/g; *p* = 0.0010). This stands in agreement with Andréasson et al., who reported higher FC values in SSc patients with GI involvement (130 vs. 57 μg/g; *p* = 0.013). In the study by Marie et al., the presence of SIBO was more frequent in the group of SSc patients with abnormal FC levels (46.2 vs. 3.1%; *p* = 0.000003) [28,29].

Marie et al. also found FC to be a useful instrument in the follow-up and eradication control in SSc patients with SIBO, observing a correlation between decreased FC levels and eradication of SIBO after three months of rotating courses of alternative antibiotic therapy in SSc patients [28]. In our study, the FC level was insignificantly lower in patients with successfully eradicated SIBO (156 vs. 137 μg/g). Nevertheless, therapy efficacy is best evaluated by symptom relief rather than breath testing and FC measures [35]. The fact that there was no significant decrease in FC values in patients with successfully eradicated SIBO and relatively high FC values after the treatment with rifaximin compared with healthy controls imply that SIBO is not independently associated with elevated FC levels in SSc patients. Approximately 90% of SSc patients present variously intensified involvement of the gastrointestinal tract followed by hypomotility and other disturbances [21]. The pathology of the intestinal wall, along with the hypomotility and stasis of the luminal content, may also lead to elevated FC levels. The limitation of our study was the lack of synchronous endoscopic assessment of the GIT of SSc patients. Therefore, the search for other underlying intestinal pathologies behind elevated FC levels in the course of SSc should be the subject of further research.

Similar to Andreasson et al., we failed to find any relationship between FC levels and patients’ GI symptoms as assessed by the abovementioned questionnaires [30]. This is in contrast to Marie et al.’s findings, which described higher median value for the global symptom score (GSS) of digestive symptoms in SSc patients with abnormal FC levels than in those without (median value of 3 vs. 2; *p* = 0.0006) [28]. The observations concerning the FC levels in SSc patients with reflux disease should exclude the possibility of GERD being independently associated with elevated FC levels and rather imply the role of SIBO in this matter. In hitherto studies, laboratory results of SSc patients with FC levels above 50 μg/g have shown higher median levels of C-reactive protein, while those of patients with FC level above 200 μg/g have shown significantly lower values of ferritin and plasma folic acid [28]. In our study, we found a positive correlation between elevated FC levels and higher neutrophils count among SSc patients with SIBO diagnosis. In SSc patients without SIBO diagnosis, a positive correlation was observed between FC level and age, CRP value, neutrophils, and leukocyte level.

Contrary to our expectations, higher FC values were associated with the presence of ACA antibodies. Studies to date have not described correlation of ACA antibodies with gastrointestinal involvement; therefore, the correlation between high FC values and ACA remains surprising [43]. As expected, FC was also a sensitive and specific marker to detect diffuse SSc subtype. Gut involvement in systemic sclerosis occurs more commonly in the diffuse than the limited variant [35].

## 5. Conclusions

FC turned out to be a sensitive and specific marker differentiating patients from the control and study group (SSc detection). Additionally, this marker showed high sensitivity and specificity in detecting SIBO in SSc patients. Therefore, we find FC a valuable diagnostic tool, which may enhance the validity of LHBT. Nevertheless, FC alone is not sufficient to detect SIBO. On the basis of our research, we find FC a helpful tool in monitoring the course of the disease in SSc patients and reflecting the gastrointestinal impairment and possible need for more serious and invasive gastrointestinal diagnostics.

The development of SIBO in SSc patients, when associated with intense gastrointestinal symptoms and frequent diarrhoea often leads to malabsorption and malnutrition. This puts SSc patients at risk of further complications, disease progression and morbidity and mortality rates increase. Therefore the diagnostic approach and management of SSc gastrointestinal manifestations should remain active areas of research.

## Figures and Tables

**Table 1:** Characteristics of the study and control groups including SIBO and fecal calprotectin levels.

Variable	Study Group	Absence of SIBO ^1^	Presence of SIBO ^1^	*p*	Fecal Calprotectin Levels [ug/g] *Me*		*p*	
**Gender** Female Male	39 (97.5%) 1 (2.5%)	20 (95.2%) 1 (4.8%)	19 (100%) -	0.9596	118 20		-	
**Disease subtype** dcSCC ^2^ IcSCC ^3^	6 (15.4%) 33 (84.6%)	2 (9.52%) 19 (90.48%)	4 (22.2%) 14 (77.8%)	0.5153	221.00 74		0.0943	
**Antibodies** ACA ^4^ Scl-70 ^5^ Not specified ACA ^4^/Scl-70 ^5^	22 (59.5%) 12 (32.4%) 1 (2.7%) 2 (5.4%)	12 (60%) 7 (35%) - 1 (5%)	10 (58.8%) 5 (29.4%) 1 (5.9%) 1 (5.9%)	0.7338	72 206 227 717		0.0729	
**Gastrointestinal comorbidities** No Yes	24 (60%) 16 (40%)	12 (57.1%) 9 (42.9%)	12 (63.2%) 7 (36.8%)	0.9485	96.5 119		0.2227	
**Gastrointestinal comorbidities** GERD ^6^ Esophagal hiatal hernia Others (i.a. mechanical bowel obstruction)	5 (31.3%) 7 (43.7%) 4 (25%)	3 (33.3%) 4 (44.4%) 2 (22.2%)	2 (28.6%) 3 (42.9%) 2 (28.6%)	0.9539	162 66.5 380		0.3878	
**SIBO ^1^ presence** No Yes	21 (52.5%) 19 (47.5%)	21 (100%) -	- 19 (100%)	-	24 206		0.0010	
**SIBO ^1^ eradication** No Yes	4 (26.7%) 11 (73.3%)	-	4 (26.7%) 11 (73.3%)	-	^7^ 345 156	^8^ 311 137	^7^ 0.1059	^8^ 0.1198
**8Characteristics of the control group**								
**Variable**	**Control group**	**Absence of SIBO**	**Presence of SIBO**	***p***	**Fecal calprotectin levels [ug/g]** ***Me***		***P***	
**Gender** Female Male	36 (92.3%) 3 (7.7%)	31 (91.2%) 3 (8.8%)	5 (100%) -	0.8357	20 33		0.1617	

^1^ SIBO: small intestinal bacterial overgrowth; ^2^ dcSCC: diffuse cutaneous systemic sclerosis (SSc) type; ^3^ IcSCC: limited cutaneous SSc type; ^4^ ACA: anticentromere antibodies; ^5^ Scl-70: antibodies against topoisomerase I; ^6^ GERD: gastroesophageal reflux disease; ^7^ first lactulose hydrogen breath test (LHBT) measurement before rifaximin treatment; ^8^ second LHBT measurement after rifaximin treatment.

**Table 2:** Spearman’s rank correlations between fecal calprotectin levels and selected demographic, clinical, and laboratory variables depending on SIBO presence.

Variable	Study Group					
	SIBO Absence			SIBO Presence		
	*n*	rho	*p*	*n*	rho	*p*
Age (years)	19	0.505	0.0275	17	−0.006	0.9813
Weight (kilograms)	19	−0.143	0.5592	17	0.215	0.4076
BMI ^1^	19	−0.150	0.5387	17	0.140	0.5928
Disease duration (years)	19	0.093	0.7038	17	0.336	0.1873
TP ^2^ (g/dL)	6	0.395	0.4387	4	−0.400	0.6000
Albumins (% TP)	6	−0.273	0.6004	4	0.200	0.8000
ESR ^3^ (mm/h)	16	0.208	0.4405	15	−0.075	0.7905
AST ^4^ (IU/L)	19	−0.404	0.0865	17	0.125	0.6326
ALT ^5^ (U/L)	19	−0.567	0.0114	16	0.153	0.5717
CRP ^6^ (mg/L)	19	0.564	0.0120	17	0.064	0.8078
C3 ^7^ (g/dL)	14	−0.199	0.4952	12	0.252	0.4299
C4 ^7^ (g/L)	14	−0.034	0.9072	12	−0.462	0.1309
CK ^8^ (IU/L)	3	−0.500	0.6667	10	−0.122	0.7379
Rheumatoid factor (IU/mL)	5	−0.344	0.5707	7	−0.079	0.8666
NT-proBNP (pg/mL)	14	0.474	0.0866	13	0.319	0.2886
Leukocytes (K/µL)	19	0.580	0.0093	17	0.267	0.2996
Neutrophils (K/µL)	19	0.781	0.0001	17	0.561	0.0191

^1^ BMI: body mass index; ^2^ TP: total protein; ^3^ ESR: erythrocyte sedimentation rate; ^4^ AST: aspartate transaminase; ^5^ ALT: alanine transaminase; ^6^ CRP: C-reactive protein; ^7^ C3, C4: complement component 3, 4; ^8^ CK: creatine kinase.

**Table 3:** Spearman;s rank correlations of fecal calprotectin levels and selected demographic, clinical, and biochemical variables.

Variables	Fecal Calprotectin Levels		
	*n*	rho	*p*
Age (years)	36	0.180	0.2940
Weight (kilograms)	36	−0.016	0.9257
BMI ^1^	36	−0.063	0.7138
Disease duration (years)	36	0.250	0.1413
TP ^2^ (g/dL)	10	−0.018	0.9596
Albumins (% TP)	10	0.178	0.6229
ESR ^3^ (mm/h)	31	0.144	0.4407
CRP ^4^ (mg/L)	36	0.331	0.0483
C3 ^5^ (g/dL)	26	0.131	0.5241
C4 ^5^ (g/L)	26	−0.198	0.3317
CK ^6^ (IU/L)	13	0.011	0.9715
Rheumatoid factor (IU/mL)	12	−0.212	0.5082
NT-proBNP (pg/mL)	27	0.370	0.0578
FC ^7^ (after eradication)	8	0.623	0.0991

^1^ BMI: body mass index; ^2^ TP: total protein; ^3^ ESR: erythrocyte sedimentation rate; ^4^ CRP: C-reactive protein; ^5^ C3, C4: complement component 3, 4; ^6^ CK: creatine kinase; ^7^ FC: fecal calprotectin.

**Table 4:** The clinical value of fecal calprotectin in detecting SIBO, predicting eradication efficacy, and differentiating different clinical characteristics.

Fecal Calprotectin	Sensitivity	Specificity	Criterion	AUC (95%CI)	*p*
**Group** Study vs. Control	69.44%	87.18%	>60	0.83 (0.73–0.91)	<0.0001
**SIBO ^1^ presence** Study vs. Control	76.47%	80%	>119	0.81 (0.59–0.94)	0.0054
**SIBO ^1^ (study group)** Yes No	94.12%	73.68%	>72	0.82 (0.66–0.93)	<0.0001
**Eradication (study group)** Yes No	90%	75%	≤254	0.80 (0.51–0.96)	0.0509
**Disease subtype** dcSCC ^2^ IcSCC ^3^	75.86%	66.67%	≤162	0.72 (0.54–0.86)	0.0477
**Antibodies type** ACA ^4^ Scl-70 ^5^, unknown, ACA ^4^/Scl-70 ^5^	90%	61.54%	≤162	0.74 (0.56–0.88)	0.0096
**Antibodies type** Scl-70 ^5^ ACA ^4^, unknown, ACA ^4^/Scl-70 ^5^	54.55%	86.36%	>206	0.66 (0.48–0.82)	0.1131
**Gastrointestinal diseases** Yes No	66.67%	75%	>152	0.68 (0.47–0.85)	0.2076

^1^ SIBO: small intestinal bacterial overgrowth; ^2^ dcSCC: diffuse cutaneous SSc type; ^3^ IcSCC: limited cutaneous SSc type; ^4^ ACA: anticentromere antibodies; ^5^ Scl-70: antibodies against topoisomerase I.
